# Quantitative Methylation Profiles for Multiple Tumor Suppressor Gene Promoters in Salivary Gland Tumors

**DOI:** 10.1371/journal.pone.0010828

**Published:** 2010-05-26

**Authors:** Megan L. Durr, Wojciech K. Mydlarz, Chunbo Shao, Marianna L. Zahurak, Alice Y. Chuang, Mohammad O. Hoque, William H. Westra, Nanette J. Liegeois, Joseph A. Califano, David Sidransky, Patrick K. Ha

**Affiliations:** 1 Department of Otolaryngology-Head and Neck Surgery, Johns Hopkins Medical Institution, Baltimore, Maryland, United States of America; 2 Division of Oncology Biostatistics, Department of Oncology, Johns Hopkins Medical Institution, Baltimore, Maryland, United States of America; 3 Department of Pathology, Johns Hopkins Medical Institution, Baltimore, Maryland, United States of America; 4 Department of Oncology, Johns Hopkins Medical Institution, Baltimore, Maryland, United States of America; 5 Milton J. Dance, Jr. Head and Neck Center, Greater Baltimore Medical Center, Baltimore, Maryland, United States of America; Roswell Park Cancer Institute, United States of America

## Abstract

**Background:**

Methylation profiling of tumor suppressor gene (TSGs) promoters is quickly becoming a powerful diagnostic tool for the early detection, prognosis, and even prediction of clinical response to treatment. Few studies address this in salivary gland tumors (SGTs); hence the promoter methylation profile of various TSGs was quantitatively assessed in primary SGT tissue to determine if tumor-specific alterations could be detected.

**Methodology:**

DNA isolated from 78 tumor and 17 normal parotid gland specimens was assayed for promoter methylation status of 19 TSGs by fluorescence-based, quantitative methylation-specific PCR (qMSP). The data were utilized in a binary fashion as well as quantitatively (using a methylation quotient) allowing for better profiling and interpretation of results.

**Principal Findings:**

The average number of methylation events across the studied genes was highest in salivary duct carcinoma (SDC), with a methylation value of 9.6, compared to the normal 4.5 (p<0.0003). There was a variable frequency and individual methylation quotient detected, depending on the TSG and the tumor type. When comparing normal, benign, and malignant SGTs, there was a statistically significant trend for increasing methylation in *APC*, *Mint 1*, *PGP9.5*, *RAR-β*, and *Timp3*.

**Conclusions/Significance:**

Screening promoter methylation profiles in SGTs showed considerable heterogeneity. The methylation status of certain markers was surprisingly high in even normal salivary tissue, confirming the need for such controls. Several TSGs were found to be associated with malignant SGTs, especially SDC. Further study is needed to evaluate the potential use of these associations in the detection, prognosis, and therapeutic outcome of these rare tumors.

## Introduction

Salivary gland tumors (SGTs) represent a diverse group of tumor types with a wide range of biological behaviors and histopathologic characteristics, which complicates their diagnosis and management [1], [2]. Approximately 40% of these tumors are malignant, and SGTs comprise about 5% of all head and neck malignancies, which equals only 0.3% of all malignant neoplasms [2], [3], [4].

The variable nature of SGTs creates difficulty in determining prognosis. Outcomes for patients with SGTs depend on the site of tumor, histology, extent of disease, completeness of surgery, and/or adjuvant radiation therapy, though there are many exceptions [5]. The classic example is adenoid cystic carcinoma where, despite thorough resection, up to 60% of patients experience locoregional or distant metastases. The median survival in the presence of distant metastases is around three years, though surprisingly, up to 10% of these patients may survive 10 years or longer with their metstases [6]. Thus, the behavior of this malignancy is quite variable and leads to much uncertainty for the patients afflicted with this disease. Even the most common ‘benign’ salivary tumor, pleomorphic adenoma, has a propensity for malignant transformation and possible recurrence.

The pathogenesis of human cancer is a heterogeneous process involving several pathways, and it has been proposed that the genotype may affect the clinical behavior and prognosis of the tumor [7], [8]. Recent studies have shown that development and progression of human malignancy is associated with accumulation of alterations in tumor suppressor genes (TSGs) and proto-oncogenes, and this appears to be true for some SGTs [9], [10], [11].

It is well documented that epigenetic alterations, such as DNA methylation and histone acetylation are important factors in human carcinogenesis. Methylation of cytosines in cytosine-guanine (CpG) islands contained within gene promoters can lead to transcriptional inactivation by blocking the RNA polymerase complex from binding to the promoter region. Inactivation of TSGs by hypermethylation of these CpG islands is a common feature of human carcinogenesis across many tumor types as it is associated with a partial or complete transcriptional block [12]. Methylation profiling of TSGs is quickly becoming a powerful diagnostic tool for the early detection, prognosis, and even prediction of clinical response to treatment of various cancers [13].

The etiology of most SGTs has not been determined, and little is known about the epigenetic alterations occurring within this class of neoplasms. Previous studies looking at the clinical significance of TSG promoter hypermethylation in SGTs used non-quantitative MSP and focused on a limited number of genes. There is only one other quantitative study done by Lee *et al.* that looked at promoter hypermethylation in salivary gland carcinomas using pyrosequencing, but surveyed the methylation status of only 3 genes [14]. In addition, the results from these studies have not been reproduced in a second independent study [15], [16], [17], [18]. Thus, there is a real need for more clarity regarding the association of TSG hypermethylation and SGTs, as this could lead to functional studies that could further elucidate the biology of this group of neoplasms [7]. An improved understanding of the molecular alterations associated with salivary gland tumors has the potential to improve the diagnosis, management, and outcomes seen in this patient population.

In this study we used quantitative MSP to determine the methylation frequencies and quantitative methylation values of nineteen known tumor suppressor genes in 78 SGT patients and 17 normal parotid tissue samples. Quantitative MSP has been successfully used in other tumor models and has the benefit of providing accurate and precise data regarding the level of methylation in the various tumors. Six of the genes we evaluated, including *cyclin-dependent kinase inhibitor 2A (p16)*, *stratifin 14-3-3σ*, *RAS-associated domain family protein 1A (RASSF1A)*, *retinoic acid receptor β (RAR-β)*, *death-associated protein kinase (DAPK)*, and *O^6^-methylguanine-DNA-methyltransferase (MGMT)*, were previously shown to be aberrantly methylated in salivary gland cancer [5], [7], [8], [11], [18], [19], [20]. The remaining thirteen genes we studied have been implicated in other cancer types; these genes include *absent in melanoma-1 (AIM1)*, *adenomatous polyposis coli (APC)*, *β-catenin*, *deleted in colorectal carcinoma (DCC)*, *fragile histidine triad (FHIT)*, *glutathione S-transferase P1 (GSTP1)*, *hypermethylated in cancer-1 (HIC1)*, *methylated in tumor-1 (Mint1)*, *mismatch repair protein (MLH1)*, *protein gene product 9.5 (PGP9.5)*, *thrombospondin-1 (THBS1)*, *tissue inhibitor of metalloproteinase-3 (TIMP3)*, and *target of methylation induced silencing-1 (TMS1)* [21], [22], [23], [24]. The aim of this study was to compare the promoter methylation profiles of benign SGTs (PA), and malignant SGTs (MEC, ACC, and SDC) along with normal parotid gland tissue in order to better understand the role of epigenetic silencing in salivary gland tumorigenesis.

## Materials and Methods

### Tissue samples

Tumor samples from 17 normal salivary gland specimens, and 78 paraffin embedded tumor specimens were obtained from patients surgically treated at the Department of Otolaryngology-Head and Neck Surgery (Johns Hopkins Medical Institution, Baltimore, MD, USA) using appropriate written informed consent obtained after approval by the Johns Hopkins Institutional Review Board. There were a total of 26 benign tumor samples and 52 malignant tumor samples. All 26 benign samples were pleomorphic adenomas (PA). The malignant samples included 17 adenoid cystic carcinomas (ACC), 18 salivary ductal carcinomas (SDC), and 17 mucoepidermoid carcinomas (MEC).

### DNA extraction

Paraffin embedded tissues sections were made and microdissected to ensure tumor purity. After de-paraffinization by xylene treatment, total genomic DNA was extracted by digestion with 50 µg/ml proteinase K (Boehringer, Mannheim, Germany) in the presence of 1% SDS at 48°C overnight, followed by phenol/chloroform extraction and ethanol precipitation. Genomic DNA was eluted in low-salt Tris-EDTA (LoTE) buffer and stored at −20°C.

### Bisulfite Treatment

DNA from primary tumors and normal controls were subjected to bisulfite treatment, which modifies CpG islands including those of TSG promoters, using the Epitect Bisulfite Kit from Qiagen (Valencia, California). The bisulfite-modified genomic DNA was resuspended in 120–150 uL of H_2_O and stored at −80°C.

### Quantitative Methylation-specific PCR (qMSP)

The bisulfite-modified DNA was used as a template for fluorescence-based real-time polymerase chain reaction (PCR), as previously described [25]. In brief, we evaluated the promoter methylation profiles of TSGs by fluorescence-based quantitative methylation-specific PCR (qMSP). Methylation specific primers and probes were designed to specifically amplify the bisulfite-modified promoters of the gene of interest (Table 1).

**Table 1:** Primer and probe sequences for candidate tumor suppressor genes.

Gene	Forward primer sequence (5′-3′)	Reverse primer sequence (5′-3′)	Probe sequence (5′Fam-3′Tamra)
Aim1	CGC GGG TAT TGG ATG TTA GT	CCG ACC CAC CTA TAC GAA AA	GGG AGC GTT GCG GAT TAT TCG TAG
APC	GAA CCA AAA CGC TCC CCA T	TTA TAT GTC GGT TAC GTG CGT TTA TAT	CCC GTC GAA AAC CCG CCG ATT A
β-catenin	GGA AAG GCG CGT CGA GT	TCC CCT ATC CCA AAC CCG	CGC GCG TTT CCC GAA CCG
DAP-K	GGA TAG TCG GAT CGA GTT AAC GTC	CCC TCC CAA ACG CCG A	TTC GGT AAT TCG TAG CGG TAG GGT TTG G
DCC	TTG TTC GCG ATT TTT GGT TTC	ACC GAT TAC TTA AAA ATA CGC G	GCG CTA AAC AAA AAA ACT CCG AAA A
FHIT	GGG CGC GGG TTT GGG TTT TTA C	GAA ACA AAA ACC CAC CGC CCC G	AAC GAC GCC GAC CCC ACT AAA CTC C
GSTP1	AGT TGC GCG GCG ATT TC	GCC CCA ATA CTA AAT CAC GAC G	CGG TCG ACG TTC GGG GTG TAG CG
HIC1	GTT AGG CGG TTA GGG CGT C	CCG AAC GCC TCC ATC GTA T	CAA CAT CGT CTA CCC AAC ACA CTC TCC TAC G
MGMT	CGA ATA TAC TAA AAC AAC CCG CG	GTA TTT TTT CGG GAG CGA GGC	AAT CCT CGC GAT ACG CAC CGT TTA CG
Mint1	ATT TTC GAA GCG TTT GTT TGG C	ACA AAA AAC CTC AAC CCC GC	GCG AAA CTC CCC TAC TCT CCA AC
MLH1	CGT TAT ATA TCG TTC GTA GTA TTC GTG TTT	CTA TCG CCG CCT CAT CGT	CGC GAC GTC AAA CGC CAC TAC G
p16	TTA TTA GAG GGT GGG GCG GAT CGC	GAC CCC GAA CCG CGA CCG TAA	AGT AGT ATG GAG TCG GCG GCG GG
PGP 9.5	CGG CGA GTG AGA TTG TAA GGT T	GAA CGA TCG CGA CCA AAT AAA TAC	TTC GGT CGT ATT ATT TCG CGT TGC GTA C
RAR-β	GGG ATT AGA ATT TTT TAT GCG AGT TGT	TAC CCC GAC GAT ACC CAA AC	TGT CGA GAA CGC GAG CGA TTC G
RASSF1A	GCG TTG AAG TCG GGG TTC	CCC GTA CTT CGC TAA CTT TAA ACG	ACA AAC GCG AAC CGA ACG AAA CCA
Stratifin 14-3-3σ	GAA GGT TAA GTT GGT AGA GTA GGT CGA AC	AAC TAC TAA AAA CAA ATT TCG CTC TTC G	CTC GCC CTT CTC CAC GAC GCC
THBS1	CGA CGC ACC AAC CTA CCG	GTT TTG AGT TGG TTT TAC GTT CGT T	ACG CCG CGC TCA CCT CCC T
Timp3	GCG TCG GAG GTT AAG GTT GTT	CTC TCC AAA ATT ACC GTA CGC G	AAC TCG CTC GCC CGC CGA A
TMS1	TTG GAG GGT AAC GGA TCG GGG C	CCC GCT ACA ACC GCC GAC CAA A	GAC TCC GAA ACG AAA CCT AAA CTC CCC
β-actin	TGG TGA TGG AGG AGG TTT AGT AAG T	AAC CAA TAA AAC CTA CTC CTC CCT TAA	ACC ACC ACC CAA CAC ACA ATA ACA AAC ACA

Fluorogenic PCRs were carried out in a reaction volume of 20 µL consisting of 600 nM of each primer; 200µM of probe; 0.75 U of platinum Taq polymerase (Invitrogen, Carlsbad, CA); 200 µM of each dATP, dCTP, dGTP, and dTTP; 200nM of ROX dye for reference (Invitrogen, Carlsbad, CA); 16.6 mmol/L of ammonium sulfate; 67 mmol/L of Trizma (Sigma, St Louis, MO); 6.7 mmol/L of magnesium chloride; 10 mmol/L of mercaptoethanol; and 0.1% dimethylsulfoxide. Three microliters of treated DNA solution were used in each real-time MSP reaction. Amplifications were carried out in 384-well plates in a 7900 Sequence Detector System (Perkin-Elmer Applied Biosystems, Norwalk, CT). Thermal cycling was initiated with a first denaturation step at 95°C for 2 minutes, followed by 45 cycles of 95°C for 15 seconds and 60°C for 1 minute. Leukocytes from a healthy individual were methylated in vitro with excess SssI methyltransferase (New England Biolabs Inc, Beverly, MA) to generate completely methylated DNA, and serial dilutions of this DNA were used for constructing the calibration curves on each plate. Each reaction was performed in triplicate to ensure consistent results.

The TSG promoter methylation level in each sample was calculated and normalized with respect to an internal reference gene, β-actin. This measure, which we will further refer to as methylation quotient (MQ), represents the relative level of methylation in a particular sample [(gene of interest/reference gene) ×1000] and was used for direct comparison of samples.

In order for a tumor sample to be considered methylated at a specific TSG, it had to meet two specific criteria. Amplification must have been present in at least 2 of the 3 reaction wells in the triplicate run, and the mean methylation quotient must have fallen within the range of the serial standard curve dilutions. The lack of DNA contamination was verified by the absence of amplification of a distilled water negative control for each qMSP run.

### Statistical Analysis

The primary objective in this study was to describe the methylation patterns of 19 tumor suppressor genes in normal tissue and four salivary gland tumor types: ACC, SDC, MEC and PA. To compare overall methylation levels by tumor type, the number of methylated genes for each patient was calculated as the sum of genes with any degree of methylation and the average value for normal samples was compared to the average for each tumor type using an analysis of variance.

Continuous distributions of qMSP ratios are usually skewed, often with a clump of zeros in the lower tail of the distribution. To determine if increasing methylation of any of the selected genes was seen across three categories of samples: normal, benign and malignant, two types of analyses were considered. To evaluate presence or absence of methylation for each gene, an exact version of the Cochran-Armitage trend test [26] was used to determine if the probability of methylation increased across the three categories. To evaluate the continuous methylation distributions of each gene across normal, benign and malignant categories, the non-parametric Cuzick test [27] for trend was used. All statistical computations were performed using the SAS system [28], StatXact [29] or R [30]. All p values reported are two sided.

## Results

### Clinical and Pathological Data

A total of 19 gene promoter regions were analyzed for methylation of CpG islands in 78 patients with salivary gland tumors and 17 normal salivary gland samples. The clinical and pathological characteristics of these patients, including age and smoking status, are depicted in Table 2. Of the 78 patients with salivary gland tumors, 35 were male and 43 were female and ages ranged from 12 to 85 years at the time of diagnosis. Of the 17 patients with ACC, 2 (11.8%) eventually developed local recurrence and 10 (58.8%) developed distant metastases. Of the 17 patients with MEC, 1 (5.9%) developed local recurrence and another 2 (11.8%) distant metastasis. As expected, none of the 26 patients with PA developed distant metastases, but 1 (3.8%) developed local recurrence. Of the 18 patients with SDC, 8 (44.4%) developed local recurrence and the same number developed distant metastases. The mean follow-up time for patients with malignancy (ACC, MEC, or SDC) was 3.2 years (range 0.01–18 years).

**Table 2:** Clinical and pathologic characteristics of patient populations.

Category	Subcategory	Normal	PA	ACC	MEC	SDC
**Patients, ** ***n***		17	26	17	17	18
**Age, yr, median (range)**		58.7 (42–77)	48.5 (12–74)	58 (25–83)	43 (15–77)	61 (31–85)
**Sex, ** ***n*** ** (%)**	Male	10 (58.8%)	9 (34.6%)	9 (53%)	6 (35.3%)	11 (61.1%)
	Female	7 (41.2%)	17 (65.4%)	8 (47%)	11 (64.7%)	7 (38.9%)
**Smoking Status**	Never	3 (17.6%)	13 (50%)	10 (58.8%)	11 (64.7%)	4 (44.4%)
	Former	4 (23.6%)	8 (30.8%)	4 (23.5%)	2 (11.8%)	1 (5.6%)
	Current	7 (41.2%)	5 (19.2%)	3 (17.6%)	2 (11.8%)	6 (33.3%)
	Unknown	3 (17.6%)	-	-	2 (11.8%)	3 (16.7%)
**Salivary Gland Involvement, ** ***n*** ** (%)**	Parotid		22 (84.6%)	5 (29.4%)	10 (58.8%)	16 (88.9%)
	Submandibular		1 (3.8%)	2 (11.8%)	2 (11.8%)	1 (5.6%)
	Minor		3 (11.5%)	10 (58.8%)	5 (29.4%)	1 (5.6%)
**Stage, ** ***n*** **(%)**	I		n/a	2 (11.8%)	9 (53%)	0 (0%)
	II		n/a	4 (23.5%)	4 (23.5%)	2 (11.1%)
	III		n/a	1 (5.9%)	3 (17.6%)	0 (0%)
	IV		n/a	7 (41.2%)	0 (0%)	16 (88.9%)
	Unknown		n/a	3 (17.6%)	1 (5.9%)	0 (0%)
**Local Recurrence, ** ***n*** ** (%)**	Yes		1 (3.8%)	2 (11.8%)	1 (5.9%)	8 (44.4%)
	No		25 (96.2%)	15 (88.2%)	16 (94.1%)	10 (55.6%)
**Metastasis, ** ***n*** ** (%)**	Yes		0 (0%)	10 (58.8%)	2 (11.8%)	8 (44.4%)

PA - Pleomorphic Adenoma, ACC - Adenoid Cystic Carcinoma, MEC - Mucoepidermoid Carcinoma, SDC - Salivary Ductal Carcinoma.

### Quantitative Methylation-Specific PCR in Salivary Gland Tissues

The average number of methylated genes per tissue type is shown in Table 3. As demonstrated, the number of methylated TSGs were significantly higher in SDC compared to normal salivary tissue (p<0.0003), but there was no apparent difference between normal and PA, MEC, or ACC.

**Table 3:** Average number of methylated genes per tumor type.

Tissue Type	Sample Size	Average # of Methylated Genes† (Std Dev)
**Normal**	17	4.53 (2.0)
**PA**	26	5 (2.2)
**ACC**	17	5.41 (2.4)
**MEC**	17	4.47 (2.6)
**SDC**	18	9.61* (3.0)

†Calculated as any level of methylation detected within the 19 genes tested.

*significantly different than normal, p<0.0003.

The individual methylation frequency (percentage of all samples showing some degree of methylation) and mean methylation quotient (MQ as defined previously) values for the 19 TSG loci are listed in Table 4. At least one TSG locus was methylated in 77/78 tumor samples (98.7%), and a total of 73/78 tumor samples (93.6%) showed methylation at 3 or more of the loci. The methylation frequencies varied from 0 to over 100% for different TSGs in all four tumor types, and interestingly, this was also true of the normal salivary tissue.

**Table 4:** Frequency of positive cases [n (%)] and distribution of Methylation Quotient levels [Mean (range)] in normal parotid tissue, PA, ACC, MEC, and SDC.

Gene	Normal (n = 17)		ACC (n = 17)		MEC (n = 17)		PA (n = 26)		SDC (n = 18)	
	n (%)	Mean (range)	n (%)	Mean (range)	n (%)	Mean (range)	n (%)	Mean (range)	n (%)	Mean (range)
**Aim1**	2 (11.8)	101.5 (0–1570.9)	0 (0)	0 (0-0)	0 (0)	0 (0-0)	3 (11.5)	0.9 (0–14.7)	2 (11.1)	10.3 (0–177.7)
**APC**	0 (0)	0 (0-0)	6 (35.3)	.6 (0–3.6)	2 (11.8)	0.4 (0–4.9)	9 (34.6)	39.2 (0–915.9)	15 (83.3)	1606.5 (0–9289.6)
**β-catenin**	0 (0)	0 (0-0)	0 (0)	0 (0-0)	0 (0)	0 (0-0)	0	0 (0-0)	4 (22.2)	135.9 (0–969.9)
**DAP-K**	7 (41.2)	4.8 (0–20.4)	6 (35.3)	3.2 (0–27.3)	4 (23.5)	1.8 (0–10.3)	3 (11.5)	0.5 (0–8.8)	12 (66.7)	148.1 (0–727.1)
**DCC**	0 (0)	0 (0-0)	0 (0)	0 (0-0)	0 (0)	0 (0-0)	0 (0)	0 (0-0)	0 (0)	0 (0-0)
**FHIT**	15 (88.2)	168.0 (0–309.9)	4 (23.5)	19.4 (0–103.3)	3 (17.6)	15.7 (0–116.7)	7 (26.9)	15.4 (0–86.7)	6 (33.3)	133.6 (0–1168.5)
**GSTP1**	0 (0)	0 (0-0)	0 (0)	0 (0-0)	0 (0)	0 (0-0)	0 (0)	0 (0-0)	8 (44.4)	110.9 (0–633.7)
**HIC1**	17 (100)	530.3 (182.3–1164.3)	16 (94.1)	461.6 (0–1150.7)	17 (100)	422.4 (72.8–1036.7)	26 (100)	907.8 (492.7–1843.0)	18 (100)	563.0 (162.0–1735.2)
**MGMT**	0 (0)	0 (0-0)	0 (0)	0 (0-0)	0 (0)	0 (0-0)	0 (0)	0 (0-0)	2 (11.1)	117.2 (0–1248.6)
**Mint1**	3 (17.6)	6.0 (0–37.5)	9 (52.9)	19.9 (0–111.9)	5 (29.4)	147.2 (0–2199.0)	10 (38.5)	6.4 (0–24.3)	11 (61.1)	2898.5 (0–44012.6)
**MLH1**	0 (0)	0 (0-0)	2 (11.8)	1.6 (0–20.8)	1 (5.9)	0.3 (0–4.9)	3 (11.5)	7.6 (0–128.1)	2 (11.1)	0.8 (0–8.9)
**p16**	0 (0)	0 (0-0)	0 (0)	0 (0-0)	2 (11.8)	28.3 (0–463.3)	1 (3.8)	19.8 (0–514.2)	6 (33.3)	633.1 (0–5761.5)
**PGP 9.5**	3 (17.6)	0.5 (0–4.1)	5 (29.4)	13.7 (0–156.8)	2 (11.8)	1.0 (0–13.2)	10 (38.5)	28.7 (0–274.0)	16 (88.9)	583.2 (0–2133.5)
**RAR-β**	0 (0)	0 (0-0)	3 (17.6)	8.0 (0–77.7)	4 (23.5)	342.0 (0–2934.0)	2 (7.7)	12.6 (0–313.2)	14 (77.8)	1587.2 (0–6284.7)
**RASSF1A**	0 (0)	0 (0-0)	6 (35.3)	87.6 (0–788.7)	1 (5.9)	31.8 (0–539.8)	14 (53.8)	103.6 (0–606.6)	12 (66.7)	969.7 (0–3952.4)
**Stratifin 14-3-3σ**	17 (100)	1407.8 (674.4–2439.8)	17 (100)	930.8 (0–2622.9)	17 (100)	1248.7 (0–2478.7)	26 (100)	1732.3 (607.7–6199.2)	18 (100)	1262.9 (440.1–2235.9)
**THBS1**	0 (0)	0 (0-0)	0 (0)	0 (0-0)	0 (0)	0 (0-0)	0 (0)	0 (0-0)	2 (11.1)	139.7 (0–1365.2)
**Timp3**	6 (35.3)	6.4 (0–22.7)	10 (58.8)	14.7 (0–220.2)	12 (70.6)	10.3 (0–89.4)	7 (26.9)	1.7 (0–18.3)	12 (66.7)	220.0 (0–1274.0)
**TMS1**	7 (41.2)	25.2 (0–88.9)	9 (52.9)	15.0 (0–189.8)	7 (41.2)	3.1 (0–9.4)	9 (34.6)	1.6 (0–9.1)	13 (72.2)	153.9 (0–838.6)

*Note: Frequency of positive cases is expressed as number and (%). Distribution of MQ levels is the ratio of the methylation of the gene to β-actin ×1,000.

The TSGs promoters were variably methylated in normal salivary tissue. Ten genes (*APC*, *GSTP*, *DCC*, *MLH1*, *β -Catenin*, *MGMT*, *p16*, *RAR-β*, *RASSF1A* and *THBS1*) displayed no methylation in the 17 normal tissue samples, while 2 genes (*HIC1* and *Stratifin*) showed methylation in all 17 normal tissue samples.

**Figure 1:**
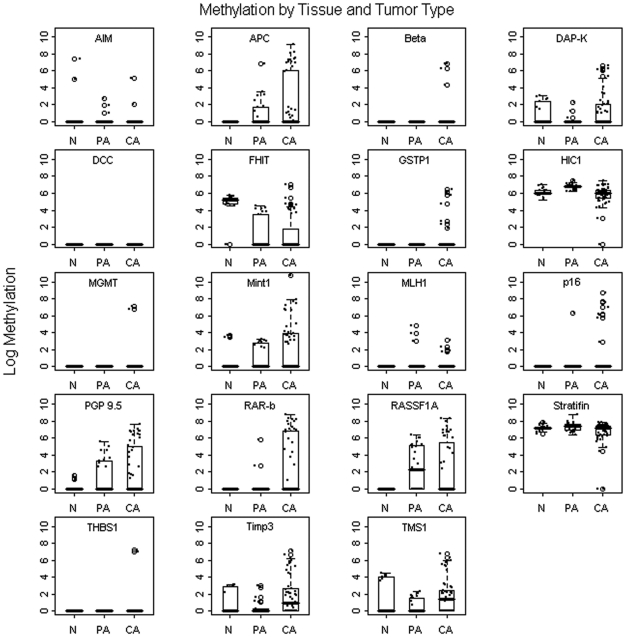
Methylation by tissue and tumor type. Overview of the log methylation quotients of normal (N), benign pleomorphic adenoma (PA), and cancerous (CA) salivary gland tumors from the 19 tumor suppressor genes tested.

The most significantly methylated tumor type was SDC, with at least 5 loci methylated in all of the 18 samples and a total of 15 (83.3%) samples methylated at 7 or more loci. The other two types of malignant tumor tested, ACC and MEC, demonstrated less methylation than SDC. For ACC, at least three loci were methylated in 15 (88.2%) of the 17 samples but only 4 (23.5%) samples were methylated at 7 or more loci. Because of the relatively small sample size and high number of variables, we could not demonstrate statistical significance in the tumor cohorts when taken individually. Figure 1 depicts a graphic illustration of the methylation quotient distribution plots by gene and tumor type.

### Methylation Levels Across Tumor Type

When dividing the groups into normal, benign (PA), and malignant (ACC, MEC, SDC), one would expect to see an increasing incidence of methylation or MQ across these three categories. The Cochran-Armitage test was used to test for increasing methylation frequency (in a binary fashion), and 7 genes met statistical significance (*APC*, *GSTP1*, *Mint1*, *P16*, *PGP 9.5*, *RAR-β*, and *Timp3*). The Cuzick test accounted for the MQ level as a continuous variable, and 8 genes met significance (*APC*, *HIC1*, *Mint1*, *PGP 9.5*, *RAR-β*, and *Timp3*). P16 and RASSF1A are interesting as they could be specific for tumors as all of the normal samples showed no methylation. However, there was methylation in benign and malignant SGTs, though they failed to meet statistical significance on both of the statistical tests (p16 was significant on the Cochran-Armitage test). Five genes showed overlapping significance in both tests (*APC*, *Mint1*, *PGP9.5*, *RAR-β*, and *Timp3*). Interestingly, *FHIT* showed a reverse correlation, with higher levels and frequency of methylation seen in the normal samples than the benign or malignant tumors. The summary data is shown in Table 5. There were no apparent correlations between any of these genes' methylation status or level and clinical or pathologic findings, including environmental factors such as age and smoking, perhaps due to the sample size limitations.

**Table 5:** Cochran-Armitage and Cuzick tests of trend across sample groups.

Gene	Normal	Benign	Carcinoma	C-A	Cuzick
		PA	ACC/MEC/SDC	p-Value	p-Value
	n (%)	n (%)	n (%)		
**Aim1**	2 (11.8)	3 (11.5)	2 (3.9)	0.19	0.54
**APC** *	0	9 (34.6)	23 (44.2)	0.002	0.006
**β-Catenin**	0	0	4 (7.7)	0.09	0.56
**DAP-K**	7 (41.2)	3 (11.5)	22 (42.3)	0.36	0.42
**DCC**	0	0	0	na	na
**FHIT** *	15 (88.2)	7 (26.9)	13 (25)	<.001	<.001
**GSTP1**	0	0	8 (15.4)	0.02	0.25
**HIC1**	17 (100)	26 (100)	51 (98.1)	0.41	0.03
**MGMT**	0	0	2 (3.9)	0.24	0.77
**Mint1** *	3 (17.7)	10 (38.5)	25 (48.1)	0.03	0.01
**MLH1**	0	3 (11.5)	5 (9.6)	0.32	0.66
**P16**	0	1 (3.9)	8 (15.4)	0.03	0.28
**PGP 9.5** *	3 (17.7)	10 (38.5)	23 (45.1)	0.05	0.03
**RAR-β** *	0	2 (7.7)	21 (40.4)	<.001	0.003
**RASSF1A**	0	14 (53.9)	19 (36.5)	0.06	0.06
**Stratifin 14-3-3σ**	17 (100)	26 (100)	50 (96.2)	0.24	0.06
**THBS1**	0	0	2 (3.9)	0.24	0.77
**Timp3** *	6 (35.3)	7 (26.9)	34 (65.4)	0.004	0.02
**TMS1**	7 (41.1)	9 (34.6)	29 (55.8)	0.15	0.28
**Total (n)**	17	26	52		

C-A: Cochran-Armitage tests for trend were used for binary methylation values.

Cuzick test for trend: a non-parametric test for continuous data values.

*statistically significant for both tests for increasing frequency in malignant tumors.

## Discussion

Promoter methylation has emerged as one of the key mechanisms of TSG silencing in many cancers, and we sought to further elucidate its role in SGTs. The aims of this study were to identify and characterize the methylation status of a broad panel of TSG promoters in several different SGTs. In order to accomplish this, we used qMSP to evaluate a panel of 19 TSGs among true SGT malignancies (ACC, MEC, SDC), one benign SGT type (PA), as well as in normal salivary tissue.

This is the first study applying qMSP to evaluate SGT promoter hypermethylation. This is also the first study to include an extensive panel of TSGs (19 in our study), whereas most of the previous studies focused only on a single gene or a small panel of genes.

Previous studies did indicate a role for hypermethylation of TSG promoters in SGT carcinogenesis. Williams *et al.* evaluated 102 tumor samples and 29 normal salivary glands using non-quantitative MSP for four TSGs (*DAPK*, *MGMT*, *RAR-β*, and *RASSF1A*). This study detected hypermethylation of *RAR-β* (29%) and *RASSF1A* (48%) for SDC and hypermethylation of *RASSF1A* (43%) for ACC [18]. In a study by Guo *et al.*, hypermethylation induced inactivation of the *p16* gene was reported in 34.2% (13 of 38) of the MEC studied [19]. Uchida *et al.* demonstrated that downregulation of *14-3-3 σ* via hypermethylation may be critical in the development of ACC. A similar study by Li *et al.*, showed promoter methylation of *p16* (47%), *RASSF1A* (41%) and *DAPK* (21%) in a cohort of 60 patients with ACC and indicated that *RASSF1A* may be linked to metastasis potential in ACC [7], [8].

Our results showed considerable heterogeneity in frequency and quantity of methylation at individual tumor suppressor genes in different SGT types. Smoking and age were not found to globally affect any methylation profiles in this cohort. We found SDC to be the most methylated tumor type, which showed significantly higher methylation frequency and level across our 19-gene panel. Interestingly, when looking at the average levels across the 19 genes tested, the normal tissue values were comparable to ACC, MEC, and PA. This finding highlights the importance of including normal controls, particularly when there are likely tissue-specific differences in methylation.

Five genes were found to have significantly increasing methylation status (frequency and level) when comparing normal, benign, and malignant salivary tissue: *APC*, *Mint1*, *PGP 9.5*, *RAR-β*, and *Timp3*. Of these, only *RAR-β* has been previously described to exhibit promoter methylation in SGTs [18]. Loss of expression of *APC*, or adenomatous polyposis coli, via promoter hypermethylation has been previously described in head and neck squamous cell carcinoma, bladder cancer, prostate cancer, lung cancer, and several other cancer types [31], [32], [33], [34]. This TSG encodes a large protein with multiple cellular functions including signal transduction in the *Wnt*-signaling pathway. In one study, there were sequence mutations of *APC* in 2/20 ACC cases, but promoter methylation was not studied [35].

*Mint1* is a protein trafficking molecule, and its methylation has been implicated in many other tumor types [36], [37], [38], [39]. The mechanism of how it suppresses growth is not known. *PGP9.5*, also known as *UCHL1* or ubiquitin carboxyl-terminal hydrolase L1, is a neuro-specific peptide that functions to remove ubiquitin from ubiquinated proteins and prevents them from targeted degradation by proteosomes [40]. This gene has also been implicated in the carcinogenesis of many tumor types, including head and neck squamous cell carcinoma, as well as pancreatic, lung, colorectal, and ovarian carcinomas [40], [41], [42], [43]. *Timp3*, or tissue inhibitor of metalloproteinases-3, has been found to inhibit angiogenesis through a VEGF mediated pathway [44], and has been found to be silenced through promoter hypermethylation in a variety of tumor types [45], [46], [47], [48]. To our knowledge, this is the first time that these three genes (*Mint1*, *PGP 9.5*, and *Timp3*) have been implicated in salivary gland tumorigenesis.

RASSF1A and p16 both seem to be specific for SGTs and not normal parotid tissue although they did not meet robust statistical significance. They could be further tested in a larger cohort for potential biomarker development for SGTs as none of the normal tissue samples showed promoter methylation. These two gene promoters may not have met statistical significance for differential methylation due to the limited samples examined and the relatively low frequency of methylation in these tissues. Previous reports have shown RASSF1A to ne hypermethylated and correlating with metastatic potential as well as tumor grade and 3-year survival [8], [14], [18].

While the significance of promoter methylation in cancer is a relatively recent discovery, it already has a wide range of possible clinical applications. It can serve as an excellent means of molecular detection of cancer in serum, saliva, and urine samples [49], [50]. In prostate cancer, promoter hypermethylation is an independent prognostic factor for relapse in cancer patients following radical prostatectomy [51]. In some instances, hypermethylation of certain TSG promoters predicts the response of tumors to therapy, as is the case with *MGMT* hypermethylation and response of primary gliomas to 1,2-bis(2-chloroethyl)-1-nitrosourea (BCNU) and temozolomide [52], [53]. It is our hope that similar findings in methylation of TSG promoters in primary SGTs might convey comparable prognostic and therapeutic implications.

In conclusion, our study is the first QMSP analysis of multiple TSGs in salivary gland tumors. The relatively high frequency and degree of methylation of some TSGs in normal salivary tissue highlights the tissue specificity of these genes, as well as the need for controlled experiments. Our results indicate that *APC*, *Mint1*, *PGP 9.5*, *RAR-β*, and *Timp3* are particularly important in SGTs, and may contribute to salivary gland carcinogenesis. Further study is needed to elucidate the mechanisms by which they contribute to tumorigenesis. Larger sample cohorts are required to determine their possible roles as markers of detection or prognosis.
